# Entropy Production from Spin–Vibrational Coupling in Endohedral-Fullerene Qubits Encapsulated in Suspended Carbon Nanotubes

**DOI:** 10.3390/e28060646

**Published:** 2026-06-08

**Authors:** Cristian Staii

**Affiliations:** Department of Physics and Astronomy, Tufts University, Medford, MA 02155, USA; cstaii01@tufts.edu

**Keywords:** endohedral fullerenes, suspended carbon nanotubes, spin–phonon coupling, Wigner function, quantum Brownian motion, entropy production, irreversibility, Lindblad dynamics, open quantum systems, quantum thermodynamics

## Abstract

Hybrid carbon nanotube–fullerene architectures provide a controllable setting in which to study irreversibility and information flow in strongly structured quantum environments. We analyze entropy generation in a platform where paramagnetic endohedral fullerenes (PEFs), such as N@C_60_ and P@C_60_, are encapsulated inside a suspended carbon nanotube (CNT) resonator, such that selected multi-level PEF spin states define an effective qubit coupled to quantized CNT flexural modes. Motivated by prior work on fullerene-filled CNTs, on spin–phonon manipulation in suspended nanotubes, and on exact phase-space propagators for damped driven oscillators, we formulate a hybrid open-system description that combines a driven quantum Brownian description of the CNT resonator with an effective Jaynes–Cummings type spin–vibrational interaction. The resonator dynamics are represented in phase space through the Wigner function, whose time evolution can be written analytically in terms of the initial Wigner distribution and a Gaussian propagator. This representation makes it possible to separate drive-induced phase space displacement, diffusion, and damping, and to connect these features directly to entropy flow. The coupled spin–mechanical dynamics are then embedded in a Lindblad quantum master equation that includes mechanical damping, spin relaxation, pure dephasing, and thermally activated excitation channels. Within this framework we derive the entropy balance equation—identifying entropy flux and non-negative entropy production—and examine how hybridization between the molecular spin and the nanotube vibration redistributes irreversibility between coherent exchange and dissipative channels. We show that spin–phonon coupling enhanced by a magnetic field gradient, resonant driving, and moderate thermal occupation can produce identifiable crossovers between entropy–production regimes dominated by the oscillator and those dominated by the spin. The resulting framework provides a quantitative basis for using CNT–PEF hybrids as nanoscale platforms for studying nonequilibrium quantum thermodynamics, decoherence, and information loss in structured vibrational environments.

## 1. Introduction

The controlled generation, storage, and degradation of quantum information in hybrid nanostructures lie at the center of both quantum technologies and nonequilibrium statistical physics [1,2,3,4,5,6,7,8,9]. Of particular interest are systems in which localized spin degrees of freedom interact with vibrational modes that are both strongly confined and experimentally accessible. In such systems, irreversibility is not merely a secondary effect but also a source of information about the pathways through which energy, coherence, and entropy are redistributed among subsystems and reservoirs [10,11,12,13,14,15,16].

Endohedral fullerenes, especially paramagnetic endohedral fullerenes (PEFs) such as N@C_60_ and P@C_60_, are especially attractive molecular spin units because they combine long spin coherence times with chemically robust carbon cages and a rich internal level structure [17,18,19]. Their encapsulation inside carbon nanotubes leads to “peapod” architectures in which the fullerenes are spatially confined, electronically addressable, and mechanically embedded in a quasi-one-dimensional (1D) host [20,21,22,23]. Such fullerene-filled nanotubes have been investigated as candidate architectures for quantum information processing, spin transport, and engineered exchange interactions [24,25,26]. In this setting, the nanotube is not merely a passive scaffold: when suspended, it supports quantized flexural modes that can couple to internal spins either intrinsically, via spin–orbit and deflection-mediated mechanisms, or extrinsically, or through externally applied magnetic-field gradients [10,11].

Suspended CNT resonators provide a particularly compelling platform for studying spin–vibrational irreversibility because they naturally combine three features. First, their flexural modes can be coherently driven and detected with high sensitivity [27,28,29,30,31,32,33]. Second, their coupling to localized states can be tuned into regimes described by effective Jaynes–Cummings or Rabi-type models [12,34,35,36]. Third, their interaction with phononic, electronic, and electromagnetic environments can be captured within open-system formalisms that expose entropy flow at the quantum level [37,38,39,40,41]. Previous work on suspended CNT spin qubits has also shown that mechanical motion can mediate electrically controllable spin resonance and coherent spin manipulation [10,11]. At the same time, exact phase space methods developed for the damped driven quantum oscillator provide a transparent way to treat the mechanical mode in terms of its Wigner function, with diffusion, damping, and forcing entering as distinct ingredients of the propagator [42,43,44].

In recent work, our group developed a unified all-mechanical protocol for coherent control and quantum-state reconstruction of suspended CNT resonators operated in the anharmonic regime [45]. Using a nearby AFM tip as a single localized actuator, we showed that the fundamental flexural mode can be driven through Rabi and Ramsey sequences, thereby allowing direct extraction of the relaxation and coherence times $T_{1}$ and $T_{2}$, while the same actuator can implement controlled phase space displacements for Wigner function tomography via displaced-parity measurements. The analysis also identified the conditions under which regions with negative Wigner function and other nonclassical signatures remain observable and provided realistic parameter estimates showing that such measurements are feasible in cryogenic CNT devices with present-day experimental capabilities.

The present work builds on these developments to address entropy production in a hybrid system composed of PEF spin qubits encapsulated in a suspended CNT resonator (the PEF-CNT system). Our aim is to construct a physically grounded analytical framework that links four elements: (i) the PEF-CNT architecture and its spin content; (ii) the driven, damped motion of the suspended CNT; (iii) the effective spin–phonon coupling between selected PEF states and a CNT flexural mode; (iv) the entropy balance associated with the resulting Lindblad dynamics.

The central conceptual point is that the CNT mechanical mode acts simultaneously as a controllable quantum subsystem and as an intermediary through which structured dissipation influences the PEF spin. In phase space, the mechanical state evolves by convolution with an exact Gaussian propagator, making visible the separate roles of reversible phase space damping, thermal broadening, and drive-induced displacement. Once coupled to the spin sector, these processes enter the entropy balance through the reduced density operator of the joint spin–mechanical system. This enables a decomposition of irreversibility into entropy flux toward the reservoirs and intrinsic entropy production associated with departures from detailed balance.

The paper is organized as follows. Section 2 summarizes the physical ingredients of PEF-filled suspended CNTs and motivates the effective qubit description. Section 3 develops the hybrid Hamiltonian and the open-system master equation. Section 4 reformulates the oscillator sector in terms of the exact Wigner propagator for driven quantum Brownian motion and shows how this solution is embedded into the coupled dynamics. Section 5 derives the entropy balance and identifies entropy flux and entropy production. Section 6 analyzes representative parameter regimes and discusses crossovers between mechanically and spin-dominated irreversibility. Section 7 discusses the implications for experiments and for nonequilibrium quantum thermodynamics in nanoscale hybrid systems. Section 8 presents the main conclusions of this work.

## 2. Physical Platform: Endohedral Fullerenes in Suspended Carbon Nanotubes

### 2.1. PEF-Filled Carbon Nanotubes as Hybrid Quantum Architectures

Paramagnetic endohedral fullerenes consist of atoms or small clusters trapped inside a fullerene cage [17,18]. In the families N@C_60_ and P@C_60_, the enclosed atom retains an unpaired electronic spin that is well shielded by the carbon cage from environmental perturbations, giving rise to narrow spin resonances and long coherence times [19,20,21,22,23]. The use of fullerene cages as molecular spin carriers has motivated extensive proposals for quantum information processing, including arrays of spin-bearing fullerenes assembled within carbon nanotubes [24].

**Figure 1:**
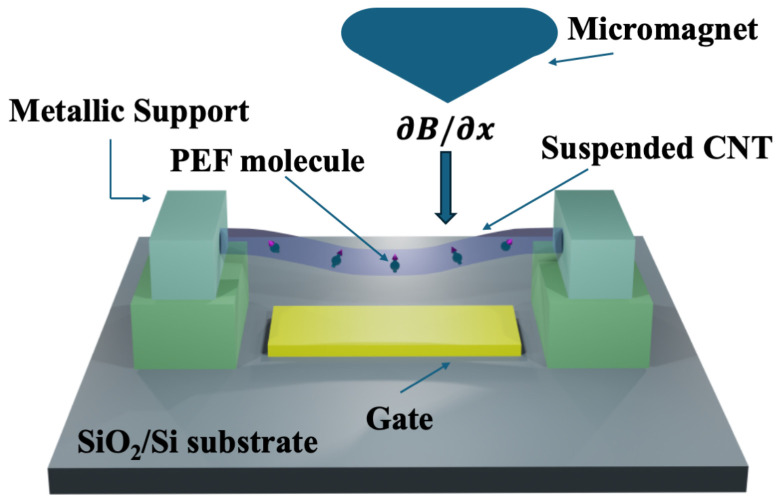
**Schematic of the proposed experimental setup showing single spins trapped inside a suspended carbon nanotube (CNT).** The spins are associated with paramagnetic endohedral fullerene (PEF) molecules confined within the CNT. The vibrational motion of the nanotube is controlled by gate voltages, while a micromagnet placed nearby generates a non-uniform external magnetic field. The figure indicates the relevant magnetic-field gradient, ∂B/∂x, where *B* is the component of the micromagnet field along the PEF spin quantization axis and *x* is the CNT flexural displacement coordinate. This field gradient makes the PEF spin splitting position dependent, giving rise to spin–vibrational coupling between the PEF spin degree of freedom and the quantized mechanical motion of the nanotube and enabling mechanical control of the PEF spin splitting.

Suspended CNT resonators provide exceptionally low effective mass, large vibrational motion, and high mechanical frequencies spanning MHz–GHz, thus enabling ground-state operation and strong coupling to spins [27,28,29,30,31,32,33,34]. When inserted into CNTs, endohedral fullerenes form ordered or quasi-ordered 1D chains in which intermolecular spacing, host–guest interactions, and nanotube confinement can all influence spin dynamics (Figure 1). Experimental and theoretical studies of fullerene peapods have highlighted several relevant features: (i) the CNT can stabilize the fullerene chain; (ii) it can modify electronic and magnetic interactions through confinement and charge redistribution; (iii) it can serve as a transport channel or an electromechanical element. For quantum-control applications, the fullerene spin can therefore be modeled as interacting with a structured environment that is intermediate between a local environment and a continuum reservoir.

In this work we focus on a suspended segment of CNT hosting several PEF molecules and concentrate fullerenes whose low-energy spin manifold defines an effective qubit. The CNT’s lowest flexural mode acts as the principal bosonic mode, while other vibrational modes and external reservoirs are treated as dissipative environments. This reduced description is appropriate when one flexural mode is tuned near resonance with a controllable spin splitting and remains spectrally well separated from the rest of the mechanical spectrum.

### 2.2. Effective Spin Manifold of Endohedral Fullerene Qubits

Both N@C_60_ and P@C_60_ exhibit high-spin electronic ground states originating from the encapsulated atom, together with hyperfine coupling to a nuclear spin [24]. For entropy-production analysis it is sufficient to project this richer manifold onto two states, {|g〉,|e〉}, which are selected by external magnetic fields and microwave-frequency addressing. The resulting splitting

(1) $$\hbar \omega_{s}=E_{e}-E_{g},$$

can be tuned by a static magnetic field, although hyperfine structure and anisotropies may generate additional nearby transitions. Our treatment therefore assumes that either those additional levels are far detuned from the mechanical mode and external drive, or they contribute only perturbatively through renormalized rates and couplings.

This effective qubit approximation is standard in hybrid spin–oscillator descriptions [10,11] and is particularly useful for identifying entropy pathways. In particular, it permits one to distinguish clearly between entropy associated with spin populations and coherences, on the one hand, and entropy associated with the vibrational phase-space distribution, on the other [44].

### 2.3. Suspended CNT Resonator and External Control

A suspended CNT behaves as a nanomechanical resonator with flexural frequencies ranging from the MHz to GHz regimes, depending on its length, tension, and electrostatic environment [27,28,29]. In the harmonic approximation, the relevant mechanical mode is described by the Hamiltonian [45]

(2) $$H_{m}=\hbar \omega_{m}a^{†}a,$$

where $\omega_{m}$ is the angular frequency of the CNT flexural mode, and *a* and $a^{†}$ are bosonic annihilation and creation operators. Coherent driving can be implemented electrically or magnetically by applying, for example, an ac gate voltage that produces a time-dependent force on the CNT flexural coordinate (Figure 1) [27,28]. Writing the mechanical displacement operator as $\hat{x}=x_{\mathrm{zpf}}\left(a+a^{†}\right)$, where $x_{\mathrm{zpf}}$ is the zero-point displacement amplitude, the laboratory-frame drive Hamiltonian may be written as [10,45]

(3) $$H_{d}^{\mathrm{lab}}\left(t\right)=-F_{d}\left(t\right)\hat{x}=-F_{0}x_{\mathrm{zpf}}\left(a+a^{†}\right)\cos\left(\omega_{d}t\right),$$

where $F_{d}\left(t\right)=F_{0}\cos\left(\omega_{d}t\right)$ is the externally applied drive force acting on the CNT flexural coordinate.

Following earlier work on suspended CNT spin qubits, spin–motion coupling can arise when the nanotube displacement modulates the local magnetic field seen by the spin or alters a spin–orbit mediated quantization axis [10,11]. In the fullerene setting considered in Figure 1, an externally imposed magnetic field gradient is especially natural: if the fullerene spin is displaced together with the nanotube, then its Zeeman splitting becomes position dependent. Quantizing the displacement yields a spin–phonon interaction linear in the oscillator coordinate. Near resonance and after a rotating-wave approximation, this reduces to a Jaynes–Cummings type coupling, as we show in the next section.

## 3. Hybrid Open-System Model

### 3.1. System Hamiltonian

The total Hamiltonian is written as

(4) $$H\left(t\right)=H_{s}+H_{m}+H_{\mathrm{int}}+H_{d}^{\mathrm{lab}}\left(t\right),$$

with

(5) $$H_{s}=\frac{\hbar \omega_{s}}{2}\sigma_{z},\;H_{m}=\hbar \omega_{m}a^{†}a,$$

and with the effective spin–phonon interaction:

(6) $$H_{\mathrm{int}}=\hbar g_{1}\left(a+a^{†}\right)\sigma_{x},$$

where $g_{1}$ is the displacement-mediated coupling strength. Here the Pauli operators are defined on the {|g〉,|e〉} manifold as $\sigma_{z}=\left|e\rangle \;\langle e\right|-\left|g\rangle \;\langle g\right|$ and $\sigma_{x}=\left|g\rangle \;\langle e\right|+\left|e\rangle \;\langle g\right|$.

Using $\sigma_{x}=\sigma_{+}+\sigma_{-}$, one may decompose $H_{\mathrm{int}}$ into resonant and counter-rotating contributions. Close to resonance, $\omega_{s}\approx \omega_{m}$, and for $g_{1}\ll \omega_{s},\omega_{m}$, the rotating-wave approximation (RWA) neglects the rapidly oscillating counter-rotating terms $a\sigma_{-}$ and $a^{†}\sigma_{+}$. Moving to a frame rotating at $\omega_{m}$ and applying RWA yields the Jaynes–Cummings Hamiltonian (see Appendix A)

(7) $$H_{\mathrm{JC}}\left(t\right)=\frac{\hbar \Delta }{2}\sigma_{z}+\hbar g_{1}\left(a\sigma_{+}+a^{†}\sigma_{-}\right)-\frac{F_{0}x_{\mathrm{zpf}}}{2}\left(a+a^{†}\right).$$

where $\Delta =\omega_{s}-\omega_{m}$ is the detuning frequency, and $\sigma_{+}=\left|e\rangle \;\langle g\right|$, $\sigma_{-}=\left|g\rangle \;\langle e\right|$ are the spin ladder operators. Equation (7) describes coherent exchange between the fullerene qubit and the CNT phonon mode.

In practice, the coupling $g_{1}$ may contain contributions from intrinsic spin–orbit–deflection mechanisms and from externally generated magnetic gradients. A useful estimate for the gradient-induced term is [10]

(8) $$g_{1}∼\frac{g_{e}\mu_{B}}{\hbar }\left(\frac{\partial B}{\partial x}\right)x_{\mathrm{zpf}},$$

where $g_{e}$ is the electronic *g*–factor, $\mu_{B}$ the Bohr magneton, ∂B/∂x the local gradient, and $x_{\mathrm{zpf}}$ the zero-point fluctuation amplitude of the CNT flexural mode. This expression makes explicit the central design principle: increasing the magnetic gradient or softening the mechanical mode enhances the spin–vibrational hybridization.

### 3.2. Dissipative Channels and Lindblad Equation

The hybrid system interacts with external environments through both spin and mechanical channels. We model the reduced density operator ρ of the coupled spin–mechanical system with the Lindblad master equation [3,44]

(9) $$\dot{\rho }=-\frac{i}{\hbar }\left[H_{\mathrm{JC}}\left(t\right),\rho \right]+L_{m}\left[\rho \right]+L_{s}\left[\rho \right].$$

For the mechanical mode we take the dissipator term [3,10,44].

(10) $$\begin{array}{cc} L_{m}\left[\rho \right] & =\gamma_{m}\left({\bar{n}}_{m}+1\right)\left(a\rho a^{†}-\frac{1}{2}\left\{a^{†}a,\rho \right\}\right)\\ \; & \;+\;\gamma_{m}{\bar{n}}_{m}\left(a^{†}\rho a-\frac{1}{2}\left\{aa^{†},\rho \right\}\right), \end{array}$$

where $\gamma_{m}$ is the mechanical damping rate and ${\bar{n}}_{m}={\left[\exp\left(\hbar \omega_{m}/k_{B}T_{m}\right)-1\right]}^{-1}$ is the thermal phonon occupation of the mechanical reservoir.

For the spin qubit we include relaxation, thermal excitation, and pure dephasing, and the dissipator is given by [3,44,45]

(11) $$\begin{array}{cc} L_{s}\left[\rho \right]= & \Gamma_{↓}\left(\sigma_{-}\rho \sigma_{+}-\frac{1}{2}\left\{\sigma_{+}\sigma_{-},\rho \right\}\right)+\Gamma_{↑}\left(\sigma_{+}\rho \sigma_{-}-\frac{1}{2}\left\{\sigma_{-}\sigma_{+},\rho \right\}\right)\\ \; & +\frac{\Gamma_{\phi }}{2}\left(\sigma_{z}\rho \sigma_{z}-\rho \right). \end{array}$$

The rates $\Gamma_{↓}$ and $\Gamma_{↑}$ denote spin relaxation and thermal excitation, respectively, and are determined by the bath spectral density at the spin transition frequency $\omega_{s}$ [45]. The rate $\Gamma_{\phi }$ accounts for pure dephasing induced by, for example, magnetic noise or slow charge fluctuations that shift the spin splitting. In this effective description the spin rates are not derived from a single microscopic bath model. Rather, $\Gamma_{↓}$, $\Gamma_{↑}$, and $\Gamma_{\phi }$ are phenomenological rates that summarize spin relaxation, thermally activated excitation, and pure dephasing due to several possible microscopic mechanisms, including phonon mediated relaxation in the CNT/fullerene environment, magnetic field noise from the device and micromagnet, charge-noise induced shifts of the local spin splitting, and nuclear-spin or hyperfine-related spectral diffusion. They can be related to experimentally measured spin-relaxation and coherence times through $1/T_{1}=\Gamma_{↓}+\Gamma_{↑}\equiv \Gamma_{1}$, and $1/T_{2}=\left(\Gamma_{↓}+\Gamma_{↑}\right)/2+\Gamma_{\phi }\equiv \Gamma_{2}$ [3,44,45].

For thermal spin reservoirs, the excitation and relaxation rates obey detailed balance, $\Gamma_{↑}/\Gamma_{↓}=\exp\left[-\hbar \omega_{s}/\left(k_{B}T_{s}\right)\right]$, so that $\Gamma_{↑}$ is strongly suppressed at low temperature when $\hbar \omega_{s}\gg k_{B}T_{s}$ [3,44]. Literature values for endohedral fullerene spins give coherence times in the range $T_{2}∼10^{-5}$–$10^{-4}\;s$, and up to $T_{2}≃0.2$–0.3ms for *N*@*C*_60_ under favorable conditions [19,23]. Encapsulation in a CNT device can also modify the spin-lattice relaxation environment [24,46]. Therefore, in a suspended CNT device with nearby gates and a micromagnet, additional magnetic and charge noise may increase the effective dephasing rate, and the representative value $\Gamma_{2}=\Gamma_{\phi }+\left(\Gamma_{↓}+\Gamma_{↑}\right)/2∼10^{5}\;s^{-1}$ used below should be viewed as a conservative device-level estimate rather than an intrinsic fullerene limit. Equations (9)–(11) define the dynamical generator used throughout this work. They provide a minimal but flexible description of entropy generation in the driven hybrid system.

### 3.3. Reduced Equations and Weak-Coupling Structure

The full density operator contains both spin and oscillator correlations. For analytical insight, it is useful to identify two complementary limits. In the weak-hybridization regime $g_{1}\ll \gamma_{m},\Gamma_{2}$, the mechanical mode behaves as a damped driven oscillator [45] that perturbs the spin through a structured noise spectrum. In the near-resonant strong cooperativity regime $g_{1}$ competes with the dissipative rates, so that coherent excitation exchange appreciably reshapes the entropy budget. The Wigner-function formulation developed below is especially convenient in the first regime, but it also remains useful in the second as a way to visualize the oscillator’s contribution to the joint state.

## 4. Wigner-Function Description of the CNT Resonator

### 4.1. Driven Quantum Brownian Motion and Exact Propagator

In the absence of coupling to the spin, the CNT flexural mode reduces to a damped driven quantum oscillator [45]. The Wigner-function formalism provides a particularly useful phase-space representation for this mode because it encodes the full information contained in the oscillator density matrix while offering an intuitive picture of the dynamics in terms of quasi-probability flow in position–momentum space [47,48]. Unlike a classical probability distribution, the Wigner function can take negative values, thereby encoding genuinely quantum features such as interference and nonclassicality, while still allowing damping, diffusion, and coherent driving to be visualized in a form closely connected to classical trajectories [45,49,50,51]. For linear systems coupled to Gaussian environments, this formalism is especially powerful because the time evolution of the mechanical state can be expressed exactly through a Gaussian propagator, with the deterministic drift of the phase-space center and the noise-induced broadening of the distribution appearing separately and transparently [42,43]. This makes the Wigner representation particularly well suited for the present problem, where we wish to distinguish reversible transport from irreversible diffusion and damping, and later relate these features directly to entropy flow and entropy production.

In phase space, the reduced mechanical state is represented by the Wigner function [48]

(12) $$W\left(q,p,t\right)=\frac{1}{2\pi \hbar }\int_{-\infty }^{\infty }d\xi \;e^{-ip\xi /\hbar }\langle q+\frac{\xi }{2}|\rho_{m}\left(t\right)|q-\frac{\xi }{2}\rangle ,$$

where *q* and *p* denote the canonical position and momentum variables of the CNT flexural mode in phase space, and $\rho_{m}$ is the oscillator density matrix. For linear damping and Gaussian reservoirs, the Wigner function evolves under an exact Gaussian propagator of the form [42]

(13) $$W\left(x,t\right)=\int d^{2}x^{\prime }\;K_{W}\left(x,t|x^{\prime },0\right)\;W\left(x^{\prime },0\right),$$

with $x={\left(q,p\right)}^{T}$. The propagator can be written schematically as [42]

(14) $$K_{W}\left(x,t|x^{\prime },0\right)=\frac{1}{2\pi \sqrt{\det\Sigma (t)}}\exp\left[-\frac{1}{2}{\left(x-\bar{x}\left(t;x^{\prime }\right)\right)}^{T}\Sigma^{-1}\left(t\right)\left(x-\bar{x}\left(t;x^{\prime }\right)\right)\right],$$

where $\bar{x}\left(t;x^{\prime }\right)$ is the classical damped driven trajectory launched from $x^{\prime }$ and Σ(t) is the covariance matrix generated by environmental noise. This structure expresses the solution as a classical flow together with diffusive broadening [42,43].

For a harmonic oscillator of mass *m* and frequency $\omega_{m}$ with a damping kernel reducible to a Markovian rate $\gamma_{m}$, the phase-space center obeys

(15) $$\dot{q}=\frac{p}{m},\;\dot{p}=-m\omega_{m}^{2}q-\gamma_{m}p+F_{d}\left(t\right),$$

where $F_{d}\left(t\right)$ is the applied drive force (Equation (3)). In the Markovian limit of quantum Brownian motion, the first moments obey damped-oscillator equations, while the second moments form a closed linear system that can be written in covariance-matrix form,

(16) $$\dot{\Sigma }=A\Sigma +\Sigma A^{T}+D,$$

with drift matrix *A* and diffusion matrix *D* determined by the damping and bath temperature [42,44].

This exact solution is especially useful because it cleanly separates three ingredients: (i) reversible phase-space transport generated by the oscillator Hamiltonian and external drive; (ii) irreversible contraction associated with damping; (iii) noise-induced spreading governed by *D*. These ingredients map naturally onto the entropy balance discussed later in this paper. The explicit expressions for the covariance matrix Σ(t) and the mean phase-space trajectory $\bar{x}\left(t\right)$, which determine the Gaussian propagator in Equations (13) and (14), are derived in Appendix B.

### 4.2. Embedding the Propagator in the Spin–Phonon Problem

When the spin–phonon coupling is restored, the oscillator no longer evolves autonomously. Nevertheless, the exact oscillator propagator remains useful in two ways. First, in perturbative treatments, the spin can enter as a source term or as a conditioned displacement, so that the oscillator Wigner function becomes a weighted superposition of Gaussian packets associated with spin-conditioned trajectories. Second, even in the fully coupled regime, the reduced mechanical state

(17) $$\rho_{m}\left(t\right)={\mathrm{Tr}}_{s}\;\rho \left(t\right)$$

can be monitored through its Wigner function to diagnose the onset of irreversibility: phase-space broadening, loss of negativity, and displacement diffusion provide useful indicators of increasing decoherence and entropy production.

Writing the joint density operator in the spin basis,

(18) $$\rho \left(t\right)=\sum_{\alpha ,\beta \in \{g,e\}}\rho_{\alpha \beta }^{\left(m\right)}\left(t\right)⊗|\alpha \rangle \langle \beta |,$$

one can define a matrix-valued Wigner function,

(19) $$W\left(q,p,t\right)=\left(\begin{array}{cc} W_{gg}\left(q,p,t\right) & W_{ge}\left(q,p,t\right)\\ W_{eg}\left(q,p,t\right) & W_{ee}\left(q,p,t\right) \end{array}\right),$$

whose diagonal components describe oscillator phase-space distributions conditioned on the spin state, and whose off-diagonal components encode spin–mechanical coherence. The Lindblad equation induces coupled partial differential equations for these entries. In the weak-coupling limit, the diagonal components can remain approximately Gaussian, while the off-diagonal terms typically decay on a scale set by $\Gamma_{2}$ together with coupling-induced and mechanical dephasing.

Experimentally, reconstruction of the matrix-valued Wigner function would require joint spin–mechanical tomography rather than tomography of the reduced mechanical state alone. The diagonal components $W_{gg}\left(q,p,t\right)$ and $W_{ee}\left(q,p,t\right)$ could in principle be reconstructed by repeating the experiment many times, measuring the PEF spin state in the {|g〉,|e〉} basis, and sorting the subsequent mechanical tomography data according to whether the spin was found in |g〉 or |e〉 [45,49,50,51,52]. The off-diagonal components $W_{ge}\left(q,p,t\right)$ and $W_{eg}\left(q,p,t\right)$, which encode spin–mechanical coherence, would require measurements in rotated spin bases. This could be implemented using Ramsey-type π/2 pulses before spin readout, thereby mapping the real and imaginary parts of the spin coherence onto population differences in the $\sigma_{x}$ and $\sigma_{y}$ bases [45,52]. Such a protocol would therefore require independent spin initialization, coherent spin rotations, and spin-selective readout, together with mechanical displacement and parity measurement. In practice, the spin measurement would introduce backaction by projecting the joint spin–mechanical state onto a conditional mechanical state. Thus, the reconstructed Wigner matrix should be understood as an ensemble tomographic object obtained from repeated preparations, not from a single nondestructive measurement [45,48,49,50,51,52]. The entropy production analysis in the present work refers to the premeasurement open system dynamics. If the measurement process itself is included, the spin-readout backaction and information gain would constitute an additional measurement-induced entropy and information flow channel, which could be incorporated using a quantum-trajectory or generalized measurement extension of the Lindblad description [44].

### 4.3. Mechanical Entropy in Phase Space

The von Neumann entropy of the reduced mechanical state, $S_{m}=-\mathrm{Tr}\left(\rho_{m}\ln\rho_{m}\right)$, generally cannot be written exactly as a simple functional of the Wigner function because W(q,p) need not be positive. However, for a single-mode Gaussian state, the entropy is completely determined by the symplectic eigenvalue of the covariance matrix Σ [44]. Defining

(20) $$\nu =\frac{1}{\hbar }\sqrt{\det(2\Sigma )},$$

with ν≥1, one may identify an effective thermal occupation number through $\nu =2\bar{n}+1$, or equivalently $\bar{n}=\left(\nu -1\right)/2$. Because any single-mode Gaussian state is unitarily equivalent to a thermal state with this occupation number, its entropy is

(21) $$S_{m}=-\mathrm{Tr}\left(\rho_{m}\ln\rho_{m}\right)=\left(\frac{\nu +1}{2}\right)\ln\left(\frac{\nu +1}{2}\right)-\left(\frac{\nu -1}{2}\right)\ln\left(\frac{\nu -1}{2}\right).$$

Equation (21) makes the relation between the covariance matrix and the mechanical entropy explicit: for a single-mode Gaussian state, $S_{m}$ is a monotonic function of the symplectic eigenvalue ν, and hence of detΣ. Accordingly, whenever diffusion and dissipation increase detΣ, the mechanical entropy also increases.

## 5. Entropy Balance, Entropy Flux, and Entropy Production

### 5.1. Von Neumann Entropy Balance for Lindblad Dynamics

The total nonequilibrium entropy of the coupled spin–mechanical system is the von Neumann entropy

(22) S(t)=−Tr[ρ(t)lnρ(t)].

The reduced density operator evolves according to the Lindblad master equation

(23) $$\dot{\rho }\left(t\right)=L\rho \left(t\right)=-\frac{i}{\hbar }\left[H\left(t\right),\rho \left(t\right)\right]+\sum_{j}\left(L_{j}\rho L_{j}^{†}-\frac{1}{2}\left\{L_{j}^{†}L_{j},\rho \right\}\right),$$

where L is the full dynamical generator and the $L_{j}$ are the jump operators [44]. Differentiating S(t) with respect to time gives

(24) $$\frac{dS}{dt}=-\mathrm{Tr}\left[\dot{\rho }\ln\rho \right]-\mathrm{Tr}\;\left[\rho \;\frac{d}{dt}\left(\ln\rho \right)\right].$$

Since Trρ=1 at all times, the second term vanishes, so that

(25) $$\frac{dS}{dt}=-\mathrm{Tr}\left[\left(L\rho \right)\ln\rho \right].$$

The Hamiltonian part of L does not contribute to dS/dt, because the trace of the commutator term vanishes by cyclicity of the trace and the fact that ρ commutes with lnρ. Thus, the entropy change is governed entirely by the dissipative part of the evolution.

To identify the irreversible contribution, we introduce a stationary reference state $\rho_{\mathrm{ss}}$ satisfying

(26) $$L\rho_{\mathrm{ss}}=0$$

and define the quantum *relative* entropy

(27) $$S\left(\rho \left(t\right)∥\rho_{\mathrm{ss}}\right)=\mathrm{Tr}\;\left[\rho \left(t\right)\left(\ln\rho \left(t\right)-\ln\rho_{\mathrm{ss}}\right)\right].$$

Here $\rho_{\mathrm{ss}}$ denotes the stationary state reached by the driven CNT–PEF system, in the rotating-frame description, after the combined effects of coherent driving, spin relaxation, spin dephasing, and mechanical damping are included. It is therefore the actual nonequilibrium steady state of the open driven system, rather than an auxiliary global Gibbs state.

Differentiating with respect to time yields

(28) $$\frac{d}{dt}S\left(\rho ∥\rho_{\mathrm{ss}}\right)=\mathrm{Tr}\;\left[\left(L\rho \right)\left(\ln\rho -\ln\rho_{\mathrm{ss}}\right)\right],$$

which motivates the definition of the entropy-production rate

(29) $$\Pi \left(t\right)\equiv -\frac{d}{dt}S\left(\rho \left(t\right)∥\rho_{\mathrm{ss}}\right)=-\mathrm{Tr}\;\left[\left(L\rho \left(t\right)\right)\left(\ln\rho \left(t\right)-\ln\rho_{\mathrm{ss}}\right)\right].$$

Under the standard assumptions of complete positivity and stationarity, Spohn’s inequality ensures that [44]

(30) Π(t)≥0.

Thus Π(t) measures the irreversible entropy generated by the open-system dynamics. Expanding Equation (29) and using Equation (25), one obtains

(31) $$\Pi \left(t\right)=\frac{dS}{dt}+\mathrm{Tr}\left[\left(L\rho \right)\ln\rho_{\mathrm{ss}}\right].$$

This suggests defining the entropy-flux rate as

(32) $$\Phi \left(t\right)\equiv -\mathrm{Tr}\;\left[\left(L\rho \left(t\right)\right)\ln\rho_{\mathrm{ss}}\right],$$

so that the entropy balance takes the form

(33) $$\frac{dS}{dt}=\Pi \left(t\right)-\Phi \left(t\right).$$

In practice, Φ(t) can be evaluated by truncating the mechanical Hilbert space, constructing the matrix representation of L, solving $L\rho_{\mathrm{ss}}=0$ with $\mathrm{Tr}\rho_{\mathrm{ss}}=1$, and then evaluating Equation (32). For example, in the strong-drive regime of Section 6.2, this numerical procedure would use the driven Jaynes–Cummings Hamiltonian together with the mechanical and spin dissipators in Equations (10) and (11).

For thermal reservoirs, Φ reduces to the usual heat flux form when $\rho_{\mathrm{ss}}$ is Gibbsian. In the driven CNT–PEF system, however, coherent forcing and multiple baths generally imply that the relevant stationary state is not a simple global Gibbs state, making Equation (32) the more general definition. In a nonequilibrium steady state, dS/dt=0, and Equation (33) reduces to

(34) $$\Pi_{\mathrm{ss}}=\Phi_{\mathrm{ss}}.$$

### 5.2. Mode-Resolved Decomposition

Because the dissipator is the sum of spin and mechanical contributions, the entropy production may be decomposed as [44]

(35) $$\Pi =\Pi_{m}+\Pi_{s}+\Pi_{\mathrm{corr}},$$

where $\Pi_{m}$ and $\Pi_{s}$ are associated with $L_{m}$ and $L_{s}$, respectively, and $\Pi_{\mathrm{corr}}$ accounts for the fact that the logarithm of the full state does not generally decompose additively once spin–mechanical correlations develop. In the weak-correlation regime one may approximate

(36) $$\rho ≃\rho_{s}⊗\rho_{m},$$

which yields

(37) $$\Pi ≃\Pi_{s}^{\mathrm{red}}+\Pi_{m}^{\mathrm{red}}.$$

This decomposition is useful for identifying whether irreversibility is dominated by vibrational diffusion or by spin relaxation and dephasing [1].

For a thermal mechanical bath with reference state

(38) $$\rho_{m}^{\mathrm{th}}=\frac{e^{-\beta_{m}\hbar \omega_{m}a^{†}a}}{\mathrm{Tr}[e^{-\beta_{m}\hbar \omega_{m}a^{†}a}]},$$

one finds

(39) $$\Phi_{m}=-\mathrm{Tr}\left[L_{m}\rho \ln\rho_{m}^{\mathrm{th}}\right]≃\beta_{m}J_{m},$$

where $J_{m}$ denotes the rate of energy transfer from the mechanical mode to its thermal reservoir in the weak-correlation (factorized) limit. An analogous expression applies to the spin bath when $\Gamma_{↑}/\Gamma_{↓}=e^{-\beta_{s}\hbar \omega_{s}}$ [1,44].

### 5.3. Entropy Production in the Wigner Representation

For the oscillator sector, the Wigner formulation permits a semiclassical entropy analysis when the state remains Gaussian or nearly Gaussian. In the linear Markovian regime, the phase space dynamics can be written in continuity-equation form [44],

(40) $$\partial_{t}W=-\nabla \cdot \left(J_{\mathrm{rev}}+J_{\mathrm{irr}}\right),$$

where $J_{\mathrm{rev}}$ is the reversible current generated by Hamiltonian flow and $J_{\mathrm{irr}}$ is the irreversible current arising from damping and diffusion. For Gaussian states, whose Wigner functions are positive, one typically introduces the Shannon-like phase space entropy

(41) $$S_{W}=-\int dq\;dp\;W\left(q,p,t\right)\ln W\left(q,p,t\right),$$

which provides a useful coarse-grained measure of the oscillator’s entropy. Although $S_{W}$ is not, in general, identical to the von Neumann entropy, for Gaussian states it is closely related to it through the covariance matrix and therefore serves as an intuitive diagnostic of diffusion-driven irreversibility [1,3,44].

In this representation, the irreversible phase-space current can be used to express entropy production. In particular, for linear Fokker–Planck dynamics, one obtains a quadratic form involving the drift and diffusion matrices, showing that entropy is generated whenever damping-induced contraction and thermal broadening fail to balance at the instantaneous state [3,42]. The exact propagator of Section 4 therefore provides not only the state itself but also the ingredients needed to characterize the oscillator’s entropy budget.

## 6. Representative Regimes and Entropy-Production Signatures

### 6.1. Resonant Weak-Drive Regime

Consider first a near-resonant regime $\omega_{s}\approx \omega_{m}$ with weak coherent drive and low bath temperature, such that ${\bar{n}}_{m}\ll 1$ and $\Gamma_{↑}\ll \Gamma_{↓}$. If the system is initialized in |e,0〉, coherent spin–phonon exchange produces Rabi-like oscillations between |e,0〉 and |g,1〉 [45]. Here |0〉 and |1〉 denote the zero- and one-phonon Fock states of the CNT flexural mode, respectively, so that |e,0〉 and |g,1〉 represent product states of the spin and mechanical sectors within the single-excitation manifold. In the absence of dissipation this exchange is reversible, and the entropy remains low if the state is pure. Once mechanical damping and spin dephasing are included, these oscillations are damped and entropy production becomes positive.

A simple concrete example is obtained by neglecting the weak drive to leading order over a single exchange cycle and truncating the Hilbert space to the single-excitation manifold {|e,0〉,|g,1〉}. Under the resonant Jaynes–Cummings Hamiltonian, the pure state evolves as [3]

(42) $$|\psi \left(t\right)\rangle =\cos\left(g_{1}t\right)\;|e,0\rangle -i\sin\left(g_{1}t\right)\;|g,1\rangle .$$

Tracing over the spin gives the reduced mechanical state [3,44]

(43) $$\rho_{m}^{\left(0\right)}\left(t\right)=\cos^{2}\left(g_{1}t\right)\;|0\rangle \langle 0|+\sin^{2}\left(g_{1}t\right)\;|1\rangle \langle 1|,$$

so that the mechanical entropy is (Equation (22)):

(44) $$S_{m}^{\left(0\right)}\left(t\right)=-\cos^{2}\left(g_{1}t\right)\ln\left[\cos^{2}\left(g_{1}t\right)\right]-\sin^{2}\left(g_{1}t\right)\ln\left[\sin^{2}\left(g_{1}t\right)\right].$$

Equation (44) shows explicitly that the reduced mechanical entropy oscillates between 0 and ln2, vanishing at $t=n\pi /2g_{1}$ and reaching its maximum at $g_{1}t=\pi /4+\pi n/2$. This behavior reflects reversible spin–phonon entanglement generated by coherent Jaynes–Cummings dynamics, rather than irreversible entropy production.

**Figure 2:**
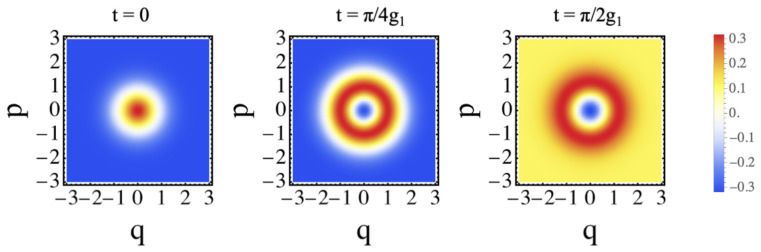
Reduced mechanical Wigner function $W_{m}\left(q,p,t\right)$ in the resonant weak-drive regime, evaluated from Equation (45) at three representative times. At t=0, the oscillator is in the vacuum state and the Wigner function is purely Gaussian. At intermediate times $t=\pi /(4g_{1})$, the phase-space distribution is a mixture of the vacuum and one-phonon contributions. At $t=\pi /(2g_{1})$, the oscillator is in the one-phonon state, and the Wigner function displays the characteristic nonclassical structure associated with a single vibrational excitation. This evolution provides a direct visualization of the coherent transfer of a single excitation from the spin sector to the CNT mode. The color bar indicates the magnitude of $W_{m}\left(q,p,t\right)$.

The corresponding reduced mechanical Wigner function also follows directly from Equation (43). Since $\rho_{m}^{\left(0\right)}\left(t\right)$ is a mixture of the vacuum and one-phonon states (see Appendix C):

(45) $$W_{m}\left(q,p,t\right)=\cos^{2}\left(g_{1}t\right)\;W_{0}\left(q,p\right)+\sin^{2}\left(g_{1}t\right)\;W_{1}\left(q,p\right),$$

where $W_{0}$ and $W_{1}$ are the Wigner functions of the harmonic-oscillator ground state and first excited state, respectively. Thus, the phase-space distribution oscillates between a purely Gaussian profile and a nonclassical one-phonon profile, providing a direct visualization of the coherent transfer of a single excitation from the spin sector to the CNT mode Figure 2.

To connect this coherent dynamics to entropy production, we now include weak dissipation perturbatively. In the low-temperature limit, the leading mechanical heat current into the bath is proportional to the instantaneous phonon occupation [44]

(46) $$J_{m}\left(t\right)≃\hbar \omega_{m}\gamma_{m}\;\langle a^{†}a\rangle =\hbar \omega_{m}\gamma_{m}\sin^{2}\left(g_{1}t\right),$$

while the corresponding entropy flux is (Equation (39)):

(47) $$\Phi_{m}\left(t\right)≃\beta_{m}J_{m}\left(t\right)=\beta_{m}\hbar \omega_{m}\gamma_{m}\sin^{2}\left(g_{1}t\right).$$

Likewise, the spin-bath channel contributes

(48) $$J_{s}\left(t\right)≃\hbar \omega_{s}\Gamma_{↓}\;\langle \sigma_{+}\sigma_{-}\rangle =\hbar \omega_{s}\Gamma_{↓}\cos^{2}\left(g_{1}t\right),$$

and therefore

(49) $$\Phi_{s}\left(t\right)≃\beta_{s}J_{s}\left(t\right)=\beta_{s}\hbar \omega_{s}\Gamma_{↓}\cos^{2}\left(g_{1}t\right),$$

where we have neglected the thermally activated spin-excitation channel $\Gamma_{↑}$.

These expressions make the redistribution of irreversibility transparent. At very short times,

(50) $$\Phi_{m}\left(t\right)\approx \beta_{m}\hbar \omega_{m}\gamma_{m}g_{1}^{2}t^{2},\;\Phi_{s}\left(t\right)\approx \beta_{s}\hbar \omega_{s}\Gamma_{↓}\left(1-g_{1}^{2}t^{2}\right),$$

showing that the mechanical entropy flux turns on quadratically as the coherent exchange transfers excitation from the spin to the CNT mode. Averaging over one Rabi period $T_{R}=\pi /g_{1}$ gives

(51) $${\bar{\Phi }}_{m}=\frac{1}{T_{R}}\int_{0}^{T_{R}}dt\;\Phi_{m}\left(t\right)=\frac{1}{2}\beta_{m}\hbar \omega_{m}\gamma_{m},\;{\bar{\Phi }}_{s}=\frac{1}{2}\beta_{s}\hbar \omega_{s}\Gamma_{↓}.$$

**Figure 3:**
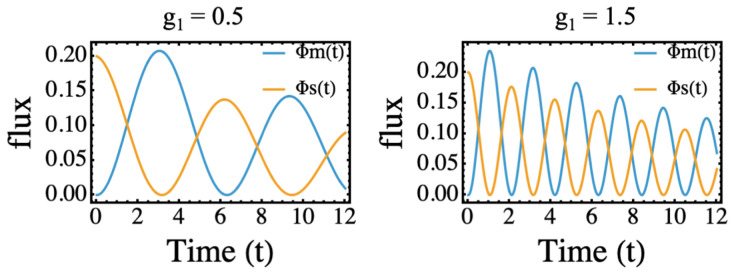
Entropy-flux dynamics in the resonant weak-drive regime for two representative values of the spin–phonon coupling $g_{1}$. The mechanical entropy flux $\Phi_{m}\left(t\right)$ and spin entropy flux $\Phi_{s}\left(t\right)$ are plotted using the weak-excitation expressions derived in the text. As $g_{1}$ increases, coherent spin–phonon exchange occurs on a shorter timescale, leading to a faster buildup of entropy flow through the mechanical channel and a corresponding redistribution of dissipation away from direct spin relaxation and toward the vibrational mode.

**Figure 4:**
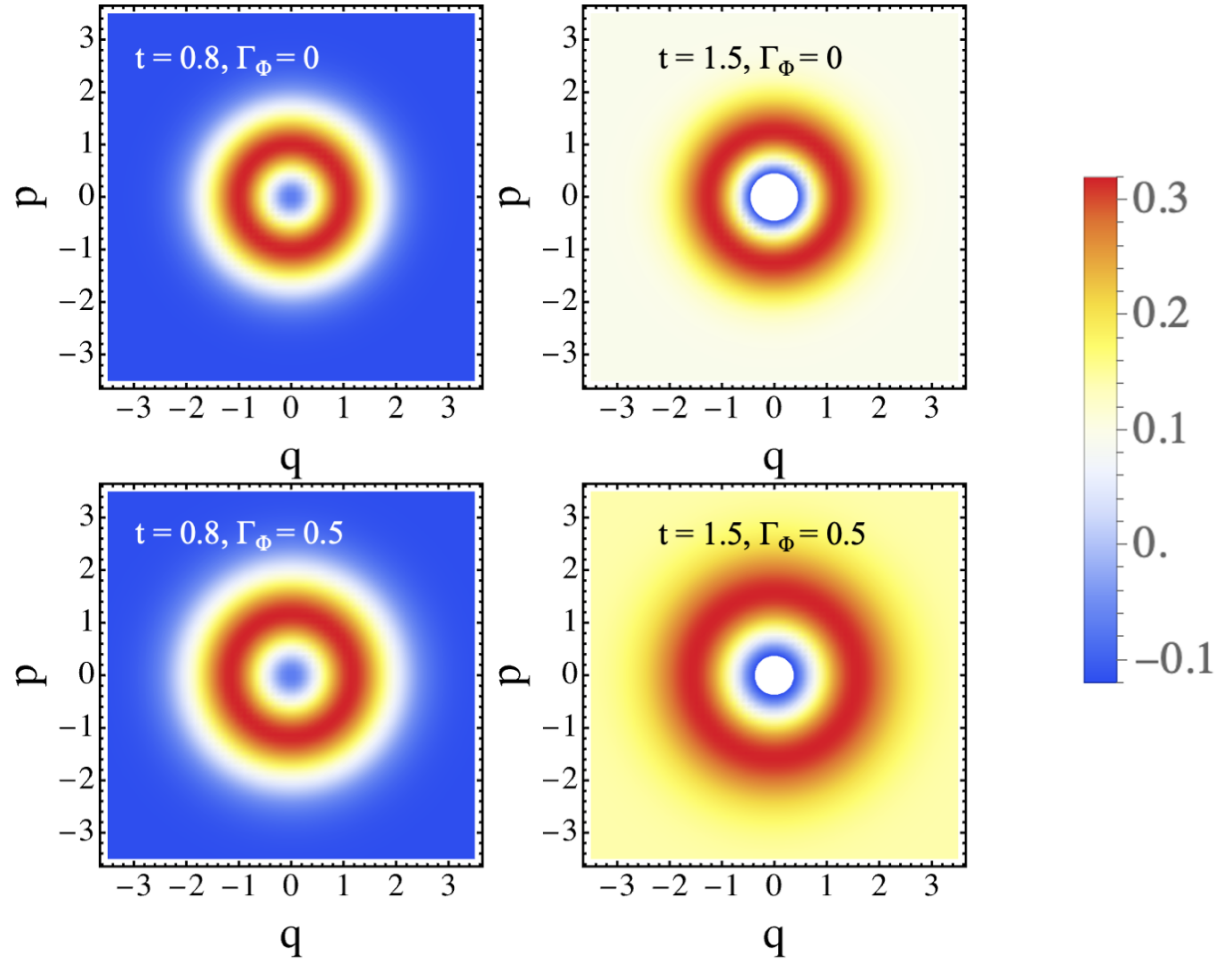
Diffusion-broadened reduced mechanical Wigner function $W_{m}\left(q,p,t\right)$ at representative times and dephasing rates in the resonant weak-drive regime. The four panels show the phase-space distribution for two times, $t=\pi /(4g_{1})$ and $t=\pi /(2g_{1})$, and for two values of the dephasing rate, $\Gamma_{\phi }=0$ and $\Gamma_{\phi }=0.5$. As dephasing increases, the nonclassical one-phonon structure is progressively smoothed and the distribution broadens in phase space, illustrating the combined effects of mechanical diffusion and spin decoherence. The color bar indicates the magnitude of $W_{m}\left(q,p,t\right)$.

**Figure 5:**
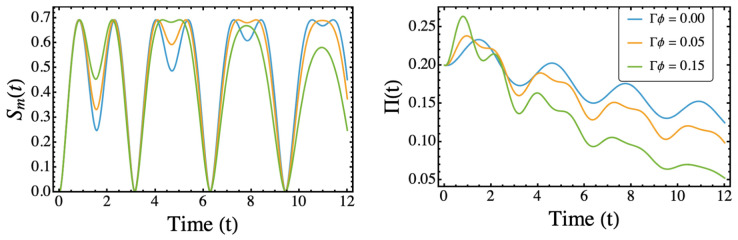
Left panel: reduced mechanical entropy $S_{m}\left(t\right)$ for three representative dephasing rates $\Gamma_{\phi }$ in the resonant weak-drive regime. Increasing dephasing progressively damps the oscillatory behavior associated with coherent spin–phonon exchange. Right panel: corresponding irreversible contribution Π(t) obtained from the phenomenological weak-excitation model. As $\Gamma_{\phi }$ increases, the entropy balance acquires a more pronounced strictly positive irreversible component arising from the combined effects of mechanical dissipation and spin decoherence. The legend shown in the inset of the right panel applies to both figures.

Thus, even in this simplest regime, the formalism (Figure 3) shows explicitly how increasing $g_{1}$ accelerates the transfer of excitation into the mechanical channel and thereby shifts part of the entropy flow from direct spin relaxation to dissipation through the vibrational mode. In the presence of weak dephasing, the oscillatory behavior in Equations (44)–(49) is progressively damped, and the Wigner function broadens (Figure 4). In addition the entropy balance acquires a strictly positive irreversible component Π(t) associated with both mechanical diffusion and spin decoherence (Figure 5).

### 6.2. Strong Driving and Phase-Space Distortion

Under resonant or near-resonant coherent drive, the oscillator acquires a large displacement, and the mechanical Wigner function is dragged along a damped orbit in phase space. In the uncoupled case the state remains Gaussian, and the entropy increase is governed primarily by the covariance matrix Σ(t). With spin–phonon coupling, the oscillator trajectory becomes spin conditioned, producing partial splitting or distortion of the Wigner distribution. These distortions reflect entangling dynamics and are accompanied by mutual information buildup between spin and oscillator.

A simple concrete example can be obtained in the rotating frame of a resonant mechanical drive, $\omega_{d}=\omega_{m}$, in the regime $\varepsilon \equiv F_{0}x_{\mathrm{zpf}}/\hbar \gg g_{1}$, where ε sets the strength of the external coherent drive acting on the oscillator (Equation (3)), and for times short compared with the spin-relaxation time, so that the spin may be treated as approximately frozen in the $\sigma_{x}$ basis. In that basis, the interaction $\hbar g_{1}\left(a+a^{†}\right)\sigma_{x}$ shifts the effective drive seen by the oscillator. If the spin is initially prepared in the state |e〉, which can be written in the $\sigma_{x}$ basis as

(52) $$|e\rangle =\frac{1}{\sqrt{2}}\left({|+\rangle }_{x}+{|-\rangle }_{x}\right),$$

and the mechanical mode starts in its ground state, then to leading order the joint state evolves into a spin-conditioned superposition of displaced oscillator states,

(53) $$|\Psi \left(t\right)\rangle =\frac{1}{\sqrt{2}}\left({|+\rangle }_{x}\;|\alpha_{+}{\left(t\right)\rangle +|-\rangle }_{x}\;|\alpha_{-}\left(t\right)\rangle \right),$$

In the above equations ${|+\rangle }_{x}$ and ${|-\rangle }_{x}$ are the eigenstates of $\sigma_{x}$, related to the {|e〉,|g〉} basis by

(54) $${|+\rangle }_{x}=\frac{1}{\sqrt{2}}\left(|e\rangle +|g\rangle \right),\;{|-\rangle }_{x}=\frac{1}{\sqrt{2}}\left(|e\rangle -|g\rangle \right).$$

In this basis the interaction is diagonal, so the strong-drive dynamics are naturally expressed in terms of spin-conditioned oscillator displacements. Here $|\alpha_{+}\left(t\right)\rangle$ and $|\alpha_{-}\left(t\right)\rangle$ are coherent states of the CNT flexural mode with spin-conditioned complex amplitudes $\alpha_{\pm }\left(t\right)$.

The coherent amplitudes obey

(55) $${\dot{\alpha }}_{\pm }\left(t\right)=-\frac{\gamma_{m}}{2}\alpha_{\pm }\left(t\right)-i\left(\varepsilon \pm g_{1}\right),\;\alpha_{\pm }\left(0\right)=0.$$

For a constant real resonant drive amplitude ε, this gives

(56) $$\alpha_{\pm }\left(t\right)=-\frac{2i}{\gamma_{m}}\left(\varepsilon \pm g_{1}\right)\left(1-e^{-\gamma_{m}t/2}\right).$$

The mean oscillator displacement is therefore large when $\left|\varepsilon \right|/\gamma_{m}\gg 1$, while the spin-dependent splitting is controlled by

(57) $$\delta \alpha \left(t\right)\equiv \alpha_{+}\left(t\right)-\alpha_{-}\left(t\right)=-\frac{4ig_{1}}{\gamma_{m}}\left(1-e^{-\gamma_{m}t/2}\right).$$

**Figure 6:**
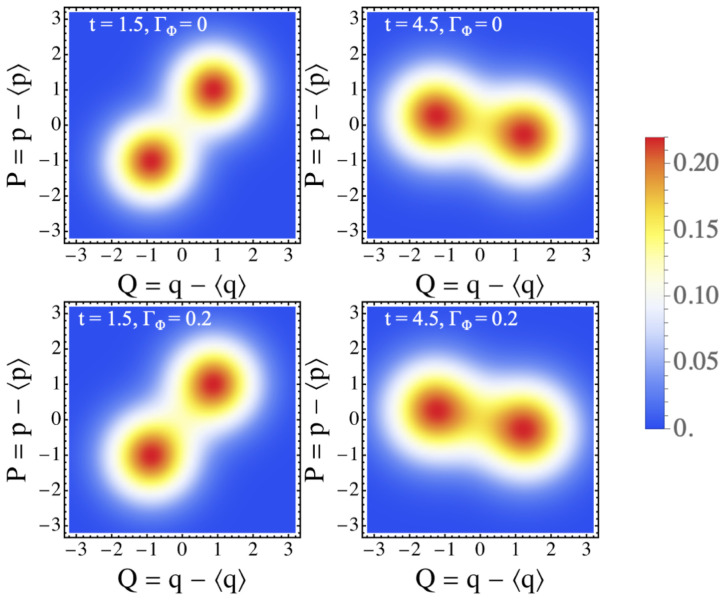
Diffusion-broadened reduced mechanical Wigner function in the strong-drive regime, shown in phase-space coordinates centered on the mean driven displacement, Q=q−〈q〉 and P=p−〈p〉. The four panels display the phase-space distribution at two representative times and for two values of the dephasing rate $\Gamma_{\phi }$. As time increases and dephasing is turned on, the two spin-conditioned Gaussian packets broaden through the covariance matrix Σ(t) and become increasingly distorted, while their overlap is reduced by decoherence. This evolution illustrates the crossover from reversible spin–oscillator entanglement dynamics to genuine irreversibility, with a growing positive entropy-production contribution associated with both finite temperature diffusion and the generation and decay of spin–oscillator correlations.

Tracing over the spin gives the reduced mechanical state

(58) $$\rho_{m}\left(t\right)=\frac{1}{2}\left(|\alpha_{+}\left(t\right)\rangle \langle \alpha_{+}\left(t\right)|+|\alpha_{-}\left(t\right)\rangle \langle \alpha_{-}\left(t\right)|\right),$$

so that the mechanical Wigner function becomes a sum of two Gaussian packets with common covariance matrix Σ(t) but different centers,

(59) $$W_{m}\left(x,t\right)=\frac{1}{2}\;G\;\left(x-{\bar{x}}_{+}\left(t\right),\Sigma \left(t\right)\right)+\frac{1}{2}\;G\;\left(x-{\bar{x}}_{-}\left(t\right),\Sigma \left(t\right)\right),$$

where

(60) $$G\left(x,\Sigma \right)=\frac{1}{2\pi \sqrt{\det\Sigma }}\exp\;\left(-\frac{1}{2}x^{T}\Sigma^{-1}x\right)$$

and ${\bar{x}}_{\pm }\left(t\right)$ are the phase-space centers associated with $\alpha_{\pm }\left(t\right)$. Equation (59) makes explicit how strong driving produces a large overall displacement, while the spin–phonon coupling generates a conditional splitting of the phase-space distribution. When the separation |δα(t)| becomes comparable to or larger than the Gaussian width set by Σ(t), the Wigner function develops a resolved double-peak structure. For smaller separations, it appears as a broadened, distorted single packet (Figure 6).

In the absence of diffusion, the entangling part of the dynamics can be quantified analytically. The overlap of the two coherent states is

(61) 〈α−(t)|α+(t)〉=exp−|δα(t)|22+iImα+α−*,

so the nonzero eigenvalues of $\rho_{m}\left(t\right)$ are

(62) $$\lambda_{\pm }\left(t\right)=\frac{1}{2}\left(1\pm e^{-{\left|\delta \alpha \left(t\right)\right|}^{2}/2}\right).$$

The reduced mechanical entropy is therefore

(63) $$S_{m}\left(t\right)=-\lambda_{+}\left(t\right)\ln\lambda_{+}\left(t\right)-\lambda_{-}\left(t\right)\ln\lambda_{-}\left(t\right).$$

This entropy grows from zero as the two spin-conditioned trajectories separate in phase space. In the purely coherent limit, Equation (63) measures reversible entanglement between the spin and the oscillator. For the pure bipartite state in Equation (53), the mutual information is

(64) $$I_{s:m}\left(t\right)=2S_{m}\left(t\right),$$

showing explicitly that phase-space splitting is accompanied by the buildup of spin–oscillator correlations.

The entropy flux to the mechanical bath can also be estimated directly. In the factorized weak-diffusion limit, the mechanical heat current is approximately

(65) $$J_{m}\left(t\right)≃\hbar \omega_{m}\gamma_{m}\;{\bar{n}}_{\mathrm{osc}}\left(t\right),$$

where the mean oscillator occupation is

(66) $${\bar{n}}_{\mathrm{osc}}\left(t\right)=\frac{1}{2}\left(|\alpha_{+}{\left(t\right)|}^{2}+{\left|\alpha_{-}\left(t\right)\right|}^{2}\right)=\frac{4(\varepsilon^{2}+g_{1}^{2})}{\gamma_{m}^{2}}{\left(1-e^{-\gamma_{m}t/2}\right)}^{2}.$$

The corresponding entropy flux is

(67) $$\Phi_{m}\left(t\right)≃\beta_{m}J_{m}\left(t\right)=\beta_{m}\hbar \omega_{m}\gamma_{m}\;{\bar{n}}_{\mathrm{osc}}\left(t\right).$$

Equations (66) and (67) show explicitly that in the strong-drive regime the entropy flux into the mechanical bath scales as $\varepsilon^{2}/\gamma_{m}$ and can become large even when the spin subsystem remains close to its local stationary state. This is the oscillator-dominated entropy-production regime.

Once diffusion and spin dephasing are restored, the two Gaussian packets in Equation (59) broaden through the covariance matrix Σ(t), while the overlap in Equation (61) is further suppressed by decoherence (Figure 6). As a result, the oscillatory entanglement dynamics cross over into genuine irreversibility: the Wigner function becomes increasingly broadened and distorted, the mutual information ceases to be purely reversible, and the entropy balance acquires a strictly positive production term Π(t) associated with both finite-temperature diffusion and the continuous generation and decay of spin–oscillator correlations.

### 6.3. Thermal Crossover and Scaling Estimates

At higher temperatures, thermal excitation of the mechanical mode increases ${\bar{n}}_{m}$ and broadens the phase space distribution, so that the fullerene qubit interacts not with a nearly pure bosonic mode but with a thermally occupied one. Two effects then follow. First, the coherent signature of spin–vibrational hybridization is progressively washed out as thermal fluctuations randomize the spin dependence. Second, the total entropy production can initially increase with temperature since a larger set of dissipative transitions becomes thermally accessible and the irreversible phase space current correspondingly grows.

When the drive injects work continuously into the system, the resulting nonequilibrium steady state typically violates global detailed balance even if each bath separately is thermal [1,44]. In that case the stationary state supports a strictly positive entropy-production rate,

(68) $$\Pi_{\mathrm{ss}}=\Phi_{\mathrm{ss}}>0,$$

because dS/dt=0 in the steady state. This quantity provides an experimentally relevant summary of irreversibility. In the present platform, $\Pi_{\mathrm{ss}}$ can be tuned by the magnetic gradient (which changes $g_{1}$, see Equation (8)), the drive amplitude, the bath temperature, and the quality factor of the suspended CNT.

A simple scaling analysis clarifies the competition between coherent hybridization and dissipation. Let the cooperativity-like ratio be defined as

(69) $$C=\frac{4g_{1}^{2}}{\gamma_{m}\Gamma_{2}}.$$

**Figure 7:**
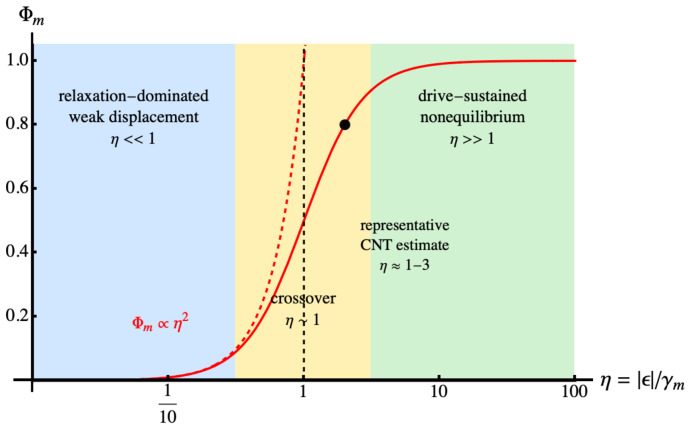
Schematic illustration of the drive-induced crossover in the mechanical entropy flux. The normalized mechanical entropy flux contribution is plotted as a function of the dimensionless drive strength $\eta =\left|\varepsilon \right|/\gamma_{m}$. The solid red curve shows a schematic crossover form, while the dashed red curve indicates the low-drive linear-response scaling $\Phi_{m}\propto \eta^{2}$. For η≪1, the system is weakly displaced and the entropy budget is relaxation dominated. Near η∼1, the system enters a crossover regime. For η≫1, the resonator is driven far from its thermal reference state and the mechanical entropy flux becomes the dominant drive-sustained contribution. The marked point indicates the representative CNT estimate η≃1–3 discussed in the text.

For C≪1, the spin and oscillator mainly relax through their own baths, and entropy production is approximately additive. For C≳1, coherent exchange competes strongly with dissipation, spin–oscillator correlations become significant, and the additive approximation fails. In this crossover regime one expects the correlation contribution $\Pi_{\mathrm{corr}}$ to become important, especially under continuous coherent driving. Introducing the dimensionless drive parameter

(70) $$\eta =\frac{\left|\varepsilon \right|}{\gamma_{m}},$$

which provides a convenient measure of drive strength $\varepsilon =F_{0}x_{\mathrm{zpf}}/\hbar$ relative to mechanical damping, one distinguishes weakly displaced states (η≪1) from strongly nonequilibrium mechanical states (η≫1). The entropy budget then crosses over from relaxation-dominated to drive-sustained, with the mechanical entropy flux scaling as $\eta^{2}$ in the linear-response regime and remaining the dominant contribution once the resonator is held far from its thermal reference state (Figure 7). We provide representative values for the parameters, which indicate that this thermal crossover should be experimentally accessible in suspended CNT devices. Using the length-dependent estimates of Ref. [45] for $Q=10^{4}$, one finds $\omega_{m}/2\pi ≃5.37\;\mathrm{GHz}$, 221MHz, and 54MHz for L=100, 500, and 1000nm, with corresponding relaxation times $T_{1}≃0.29$, 7.4, and 29.6μs and, in the relaxation-limited regime, $T_{2}≃2T_{1}$. At T=10mK these frequencies correspond to thermal occupations ${\bar{n}}_{m}≃6\times 10^{-12}$, 0.5, and 3.4, respectively, showing explicitly the crossover from a near-ground-state mechanical mode in short CNTs to a thermally occupied mode in longer devices [45].

A complementary benchmark is provided by the suspended-CNT spin–phonon estimates in Ref. [10] who obtained $g_{1}/2\pi ≃0.56\;\mathrm{MHz}$ for the spin qubit and $g_{1}/2\pi ≃0.49\;\mathrm{MHz}$ for the transverse Kramers ($K_{x}$) qubit at $\omega_{m}/2\pi ≃500\;\mathrm{MHz}$, together with a mechanical damping rate $\gamma_{m}≃5\times 10^{4}\;s^{-1}$ for $Q≃6.3\times 10^{4}$. Combining these values with a representative transverse decoherence rate $\Gamma_{2}∼10^{5}\;s^{-1}$ yields $C∼10^{4}$, i.e., well within the correlation-dominated regime. In the same work, the strong-drive response was analyzed at T=50mK with drive scale ω/2π≃0.027MHz. Identifying |ε| with this order of magnitude gives η≃ 1–3, which is consistent with a driven regime in which the mechanical entropy flux can dominate the steady-state entropy budget.

## 7. Discussion

The framework developed above brings together three ingredients that are often treated separately: molecular spin qubits based on paramagnetic endohedral fullerenes, coherently driven suspended carbon nanotube mechanics, and entropy production in open quantum systems. Their combination is compelling not only from the perspective of nonequilibrium thermodynamics but also in relation to decoherence studies, quantum information flow, and the control of hybrid quantum devices. First, PEF-filled CNTs provide a natural realization of a structured environment in which the distinction between “system” and “bath” is partially hierarchical rather than sharply binary. The selected fullerene spin is the most localized degree of freedom, and the addressed CNT flexural mode is an intermediate mesoscopic subsystem that can store, transfer, and dissipate excitations. Moreover, the remaining phononic and electromagnetic reservoirs form the broader environment. Entropy production in such a setting is richer than in a featureless Markov bath because coherent exchange, thermally activated transitions, and reservoir-induced diffusion all operate on comparable footing. This makes the platform especially relevant for studying how irreversibility emerges when a localized qubit interacts with an environment that is itself structured, controllable, and partially quantum. Second, the Wigner-function formulation is particularly well suited to this problem because the mechanical degree of freedom is both experimentally measurable and theoretically tractable. In realistic experiments one may not reconstruct the full joint density matrix of the spin–resonator system, but one can often access mechanical quadratures, sideband spectra, linewidths, and driven phase-space motion [45,47,48,49,50,51,52]. These observables constrain the covariance matrix Σ(t) and therefore the mechanical contribution to entropy generation. More broadly, the phase-space picture provides a useful bridge between quantum control and open-system thermodynamics: it makes it possible to visualize the transition from coherent state preparation to decoherence-induced broadening and, in the strong-drive regime, from reversible spin–oscillator entanglement to genuine dissipative irreversibility. Thirdly, the present framework is relevant to ongoing efforts in quantum computing and quantum information science. Hybrid spin–mechanical systems are of interest as candidate interfaces for quantum state transfer, transduction, and quantum memory, and their usefulness depends critically on understanding how coherence is degraded by vibrational and environmental channels [24,25,26]. In this context, entropy production provides more than a thermodynamic bookkeeping device: it offers a quantitative measure of how information about the spin qubit is redistributed into mechanical motion and ultimately lost to external reservoirs. The same formalism can therefore be viewed as a tool for analyzing decoherence pathways, identifying operating regimes in which coherent hybridization dominates over dissipative loss, and clarifying when a structured vibrational mode acts as a useful quantum resource rather than merely as a source of noise. From this perspective, CNT–PEF devices are relevant not only as model systems for irreversibility but also as testbeds for error mechanisms and control strategies in nanoscale quantum architectures.

The rotating-wave approximation is valid when the spin–phonon coupling is small compared with the fast frequencies that multiply the counter-rotating terms. In the interaction picture, as shown in Appendix A, the resonant terms $a\sigma_{+}$ and $a^{†}\sigma_{-}$ oscillate at the detuning $\Delta =\omega_{s}-\omega_{m}$, whereas the counter-rotating terms $a\sigma_{-}$ and $a^{†}\sigma_{+}$ oscillate at $\omega_{s}+\omega_{m}$. Thus, the Jaynes–Cummings description requires $g_{1}\ll \omega_{s}+\omega_{m}$, with the near resonant exchange regime further requiring $\left|\Delta |=\right|\omega_{s}-\omega_{m}|\ll \omega_{s},\omega_{m}$. The leading effect of the discarded counter-rotating terms is a Bloch–Siegert type shift of the resonance condition, of order $\delta_{\mathrm{BS}}∼g_{1}^{2}/\left(\omega_{s}+\omega_{m}\right)$, together with small virtual excitation corrections to the spin–mechanical eigenstates [44]. Efficient coherent exchange additionally requires |Δ| to be comparable to or smaller than the relevant coupling and linewidth scales [44,45]. These corrections become non-negligible only as the system approaches the ultrastrong-coupling regime, $g_{1}/\omega_{m}≳0.05$–0.1, or when the detuning is large enough that the near-resonant exchange picture no longer applies. In that regime, one should replace the Jaynes–Cummings Hamiltonian by the full quantum Rabi Hamiltonian, retaining the complete interaction $\hbar g_{1}\left(a+a^{†}\right)\sigma_{x}$ rather than only its excitation-conserving part. The entropy balance framework used here would remain applicable, but the quantitative entropy production rates would be modified by Bloch–Siegert shifts, virtual spin–phonon excitations, and additional correlation-mediated entropy channels. For the representative CNT parameters discussed below, however, $g_{1}/2\pi ∼0.5\;\mathrm{MHz}$ and $\omega_{m}/2\pi ∼500\;\mathrm{MHz}$, so $g_{1}/\omega_{m}∼10^{-3}$, and the Bloch–Siegert correction is expected to be negligible on the scale of the dissipative rates considered here.

The fullerene setting also provides a natural route to extensions beyond the effective two-level approximation adopted here. Both N@C_60_ and P@C_60_ possess additional spin sublevels and hyperfine structure that could support genuinely multilevel thermodynamic effects, including entropy redistribution among internal states, mode-selective relaxation pathways, and possibly autonomous refrigeration or heat engine-like cycles under suitably engineered driving protocols. The qubit treatment developed here should therefore be regarded as the lowest-order member of a broader class of spin–vibrational entropy problems in molecular nanomechanics. At the same time, several aspects of the present treatment merit further refinement. The Lindblad description assumes weak coupling to broad baths and therefore does not capture possible non-Markovian memory effects inherited from the nanotube environment, from slow magnetic fluctuations, or from additional structured vibrational modes. Likewise, the exact Gaussian propagator for the oscillator is most directly applicable for linear damping and quadratic mechanical Hamiltonians. Strong mechanical nonlinearities, intermode coupling, transport-induced backaction, or drive-induced anharmonicities would require extensions beyond the present phase space treatment. In addition, the microscopic derivation of the spin–phonon coupling for a specific fullerene species in a specific nanotube geometry remains an open materials-level problem. The description used here is therefore best viewed as an effective, physically grounded framework guided by the established physics of suspended CNT spin devices and fullerene peapods, rather than as a complete microscopic model of any one experimental realization.

These considerations also suggest several natural directions for future work. A first step is to incorporate the full hyperfine-resolved structure of N@C_60_ or P@C_60_, thereby replacing the effective qubit with a multilevel spin manifold. A second is to include multiple CNT vibrational modes and examine mode-selective entropy currents, intermode correlations, and their influence on decoherence. A third is to move beyond Markovian dissipation and analyze memory effects in the entropy balance, especially in regimes where the mechanical mode mediates long-lived backaction on the spin sector. A fourth is to connect the effective parameters of the theory more directly to experimentally realistic geometries by modeling, for example, magnetic-gradient control from a nearby nanomagnet or a magnetized AFM tip and by incorporating device-specific estimates of damping, dephasing, and thermal occupation. More generally, it would be valuable to relate entropy-production diagnostics to standard figures of merit in quantum information processing, such as state-transfer fidelity, coherence time, and gate error, thereby linking nonequilibrium thermodynamics more directly to the performance of hybrid molecular quantum devices. Taken together, these results suggest that suspended CNTs filled with paramagnetic endohedral fullerenes offer an unusually versatile setting in which to study the interplay of coherent control, decoherence, and irreversibility. They provide a platform where entropy flow can be analyzed not only as a thermodynamic quantity but also as a diagnostic of information loss and environment-induced degradation in a hybrid quantum system. In that sense, the present framework is relevant both to the foundations of nonequilibrium quantum thermodynamics and to the practical problem of understanding how structured vibrational environments shape the operation of nanoscale qubits.

## 8. Conclusions

We have presented a theoretical framework for entropy production in a hybrid quantum nanostructure composed of paramagnetic endohedral fullerene qubits encapsulated in a suspended carbon nanotube resonator. The approach combines an effective spin–phonon Hamiltonian motivated by suspended CNT spin-control schemes with a Wigner-function treatment of the driven, damped mechanical mode and an entropy-balance analysis based on Lindblad quantum dynamics.

The main result is a transparent description of how irreversibility is distributed among coherent spin–vibrational exchange, mechanical diffusion, spin relaxation, and dephasing. The Wigner representation isolates the respective roles of drive, damping, diffusion, and spin-conditioned phase space splitting in the CNT resonator, while the Lindblad entropy balance identifies the corresponding entropy flux and non-negative entropy production of the full hybrid system. This makes it possible to distinguish oscillator-dominated and spin-dominated entropy production regimes, to analyze thermal and drive-induced crossovers between them, and to identify the parameter ranges in which spin–oscillator correlations make an appreciable contribution to the entropy budget.

A further outcome of the present analysis is that it connects entropy production directly to the problem of decoherence in a structured hybrid environment. In the CNT–PEF platform, information initially stored in the localized spin qubit can be transferred coherently to the vibrational mode and then dissipated into external reservoirs. Entropy production therefore provides a quantitative way to track how coherence is degraded, how spin–oscillator correlations are generated and lost, and how a structured vibrational mode transitions from a useful quantum intermediary to a channel of irreversible information loss. More generally, the results show how phase-space methods and open-system thermodynamics can be fruitfully combined in hybrid devices where localized spins interact with controllable vibrational environments. Suspended fullerene-filled nanotubes are therefore of interest not only as experimentally relevant platforms for studying irreversibility at the single-molecule level but also as candidate architectures for quantum information processing, coherent state transfer, and hybrid quantum control. In this broader setting, entropy-production diagnostics may complement more standard quantum-information figures of merit by helping identify operating regimes in which coherent hybridization remains dominant and dissipative losses remain controllable.

Overall, these results demonstrate that PEF-filled suspended CNTs offer a versatile setting in which to study the interplay of coherent control, decoherence, and irreversibility in a structured quantum environment. They provide a platform where entropy flow can be interpreted simultaneously as a thermodynamic quantity and as a diagnostic of information redistribution and loss in a hybrid quantum device.

## Data Availability

The original contributions presented in this study are included in the article. Further inquiries can be directed to the corresponding author.

## Appendix A. Derivation of the Effective Jaynes–Cummings Hamiltonian

In this appendix we derive the effective Jaynes–Cummings (JC) Hamiltonian used in the main text for a paramagnetic endohedral fullerene (PEF) spin qubit coupled to a flexural mode of a suspended carbon nanotube (CNT). The derivation follows the logic discussed in the main text: we begin from the laboratory-frame Hamiltonian, transform to a suitable rotating frame, and then apply the rotating-wave approximation (RWA). Since the goal here is to obtain the coherent spin–phonon Hamiltonian, we do not include the environmental bath, which is treated in the main text using the Lindblad formalism.

*Laboratory-frame Hamiltonian*. The coherent PEF–CNT dynamics is described by

(A1) $$H_{\mathrm{lab}}\left(t\right)=H_{0}+H_{\mathrm{int}}+H_{d}^{\mathrm{lab}}\left(t\right),$$

with

(A2) $$H_{0}=\frac{\hbar \omega_{s}}{2}\sigma_{z}+\hbar \omega_{m}a^{†}a,$$

and

(A3) $$H_{\mathrm{int}}=\hbar g_{1}\left(a+a^{†}\right)\sigma_{x},\;H_{d}^{\mathrm{lab}}\left(t\right)=-F_{0}x_{\mathrm{zpf}}\left(a+a^{†}\right)\cos\left(\omega_{d}t\right)$$

Here $\omega_{s}$ is the PEF spin splitting, $\omega_{m}$ is the CNT mechanical frequency, *a* and $a^{†}$ are the annihilation and creation operators of the flexural mode, and $\sigma_{x},\sigma_{z}$ are Pauli operators acting in the effective two-level spin subspace. Writing

(A4) $$\sigma_{x}=\sigma_{+}+\sigma_{-},$$

the interaction term becomes

(A5) $$\begin{array}{cc} H_{\mathrm{int}} & =\hbar g_{1}\left(a+a^{†}\right)\left(\sigma_{+}+\sigma_{-}\right)\\ \; & =\hbar g_{1}\left(a\sigma_{+}+a\sigma_{-}+a^{†}\sigma_{+}+a^{†}\sigma_{-}\right). \end{array}$$

*Rotating-frame Hamiltonian*. To isolate the slowly varying near-resonant terms, we move to a frame rotating at the mechanical frequency $\omega_{m}$. We introduce the unitary transformation

(A6) $$U\left(t\right)=\exp\;\left[i\omega_{m}t\left(a^{†}a+\frac{\sigma_{z}}{2}\right)\right],$$

and define the transformed Hamiltonian as [3,44]

(A7) $$\tilde{H}\left(t\right)=U\left(t\right)H_{\mathrm{lab}}\left(t\right)U^{†}\left(t\right)-i\hbar \;U\left(t\right)\frac{d}{dt}U^{†}\left(t\right).$$

The relevant operators transform according to

(A8) $$\begin{array}{cc} UaU^{†} & =a\;e^{-i\omega_{m}t}, \end{array}$$

(A9) $$\begin{array}{cc} Ua^{†}U^{†} & =a^{†}e^{i\omega_{m}t}, \end{array}$$

(A10) $$\begin{array}{cc} U\sigma_{+}U^{†} & =\sigma_{+}e^{i\omega_{m}t}, \end{array}$$

(A11) $$\begin{array}{cc} U\sigma_{-}U^{†} & =\sigma_{-}e^{-i\omega_{m}t}. \end{array}$$

Applying Equation (A6) to the free Hamiltonian gives

(A12) $$UH_{0}U^{†}-i\hbar \;U\frac{d}{dt}U^{†}=\frac{\hbar (\omega_{s}-\omega_{m})}{2}\sigma_{z}\equiv \frac{\hbar \Delta }{2}\sigma_{z},$$

where

(A13) $$\Delta \equiv \omega_{s}-\omega_{m}$$

is the detuning frequency.

The interaction term transforms as

(A14) $$\begin{array}{cc} UH_{\mathrm{int}}U^{†} & =\hbar g_{1}\left[a\sigma_{+}+a^{†}\sigma_{-}+a\sigma_{-}e^{-2i\omega_{m}t}+a^{†}\sigma_{+}e^{2i\omega_{m}t}\right]. \end{array}$$

The first two terms are time independent in this frame and correspond to the resonant exchange of a spin excitation and a phonon. The last two terms oscillate rapidly at frequency $2\omega_{m}$ and are the counter-rotating contributions. Similarly, the drive term transforms as

(A15) $$\begin{array}{cc} UH_{d}^{\mathrm{lab}}\left(t\right)U^{†} & =-F_{0}x_{\mathrm{zpf}}\left(ae^{-i\omega_{m}t}+a^{†}e^{i\omega_{m}t}\right)\cos\left(\omega_{d}t\right)\\ \; & =-\frac{F_{0}x_{\mathrm{zpf}}}{2}[ae^{-i(\omega_{m}-\omega_{d})t}+a^{†}e^{i(\omega_{m}-\omega_{d})t}\\ \; & \;+ae^{-i(\omega_{m}+\omega_{d})t}+a^{†}e^{i(\omega_{m}+\omega_{d})t}]. \end{array}$$

For a near-resonant drive, $\omega_{d}≃\omega_{m}$, the terms oscillating at $\omega_{m}+\omega_{d}$ are rapidly varying and are neglected under the RWA. Thus

(A16) $${\tilde{H}}_{d}^{\mathrm{RWA}}\left(t\right)=-\frac{F_{0}x_{\mathrm{zpf}}}{2}\left[ae^{-i\delta_{d}t}+a^{†}e^{i\delta_{d}t}\right],$$

where $\delta_{d}=\omega_{m}-\omega_{d}$. On exact resonance, $\delta_{d}=0$, this reduces to

(A17) $${\tilde{H}}_{d}^{\mathrm{RWA}}=-\frac{F_{0}x_{\mathrm{zpf}}}{2}\left(a+a^{†}\right).$$

*System Hamiltonian in the rotating-wave approximation*. When the system is close to resonance and the coupling is weak, $\left|\Delta \right|\ll \omega_{m}$, $g_{1}\ll \omega_{m},\omega_{s}$, the rapidly oscillating terms average to zero on the timescale of the slow dynamics. Under the RWA we therefore keep only the resonant terms and obtain the Jaynes–Cummings Hamiltonian

(A18) $$H_{\mathrm{JC}}\left(t\right)=\frac{\hbar \Delta }{2}\sigma_{z}+\hbar g_{1}\left(a\sigma_{+}+a^{†}\sigma_{-}\right)-\frac{F_{0}x_{\mathrm{zpf}}}{2}\left(a+a^{†}\right).$$

Thus, the coherent spin–phonon dynamics reduces to the standard Jaynes–Cummings interaction. This is the form used throughout the main text. It shows that, near resonance, the dominant coherent process is the exchange of a single phonon in the CNT mode with a single spin excitation of the PEF qubit, with coupling strength $g_{1}$, while the detuning Δ controls the deviation from exact resonance.

## Appendix B. Gaussian Propagator and Covariance Dynamics

In this appendix, we derive the explicit Gaussian kernel entering Equation (13), together with the corresponding covariance matrix and mean-trajectory dynamics.

For a linear quantum Brownian oscillator, the Wigner function remains Gaussian if the initial state is Gaussian. Let the drift matrix in Equation (16) be

(A19) $$A=\left(\begin{array}{cc} 0 & 1/m\\ -m\omega_{m}^{2} & -\gamma_{m} \end{array}\right),$$

and let *D* denote the diffusion matrix. Then the covariance matrix evolves as

(A20) $$\Sigma \left(t\right)=e^{At}\Sigma \left(0\right)e^{A^{T}t}+\int_{0}^{t}ds\;e^{A(t-s)}De^{A^{T}\left(t-s\right)}.$$

The mean phase-space coordinate obeys

(A21) $$\bar{x}\left(t\right)=e^{At}\bar{x}\left(0\right)+\int_{0}^{t}ds\;e^{A(t-s)}f\left(s\right),$$

with $f\left(s\right)={\left(0,F_{d}\left(s\right)\right)}^{T}$. Inserting these expressions into Equation (14) yields the exact Gaussian propagator used in the main text.

## Appendix C. Wigner Function of the Reduced Mechanical State in the Weak-Drive Regime

In this appendix we derive the reduced mechanical Wigner function used in the main text for the resonant weak-drive regime. Starting from the pure Jaynes–Cummings state

(A22) |ψ(t)〉=cos(gt)|e,0〉−isin(gt)|g,1〉,

the reduced mechanical density operator obtained by tracing over the spin is

(A23) $$\rho_{m}^{\left(0\right)}\left(t\right)={\mathrm{Tr}}_{s}\;\left(|\psi (t)\rangle \langle \psi (t)|\right)=\cos^{2}\left(gt\right)|0\rangle \langle 0|+\sin^{2}\left(gt\right)|1\rangle \langle 1|.$$

Because the Wigner transform is linear in the density operator, the corresponding reduced mechanical Wigner function is

(A24) $$W_{m}\left(q,p,t\right)=\cos^{2}\left(gt\right)\;W_{0}\left(q,p\right)+\sin^{2}\left(gt\right)\;W_{1}\left(q,p\right),$$

where $W_{0}$ and $W_{1}$ are the Wigner functions of the harmonic-oscillator ground state and first excited state, respectively.

### Appendix C.1. Definition of the Wigner Function

For a mechanical density operator $\rho_{m}$, the Wigner function is defined as

(A25) $$W\left(q,p\right)=\frac{1}{2\pi \hbar }\int_{-\infty }^{\infty }d\xi \;e^{-ip\xi /\hbar }\langle q+\frac{\xi }{2}|\rho_{m}|q-\frac{\xi }{2}\rangle .$$

Applying this definition to Equation (A23) immediately gives Equation (A24) since

(A26) $$\begin{array}{cc} W_{m}\left(q,p,t\right) & =\frac{1}{2\pi \hbar }\int_{-\infty }^{\infty }d\xi \;e^{-ip\xi /\hbar }\langle q+\frac{\xi }{2}|\rho_{m}^{\left(0\right)}\left(t\right)|q-\frac{\xi }{2}\rangle \\ \; & =\cos^{2}\left(gt\right)\;\frac{1}{2\pi \hbar }\int_{-\infty }^{\infty }d\xi \;e^{-ip\xi /\hbar }\psi_{0}\;\left(q+\frac{\xi }{2}\right)\psi_{0}^{*}\;\left(q-\frac{\xi }{2}\right)\\ \; & \;+\sin^{2}\left(gt\right)\;\frac{1}{2\pi \hbar }\int_{-\infty }^{\infty }d\xi \;e^{-ip\xi /\hbar }\psi_{1}\;\left(q+\frac{\xi }{2}\right)\psi_{1}^{*}\;\left(q-\frac{\xi }{2}\right), \end{array}$$

where $\psi_{0}\left(q\right)=\langle q|0\rangle$ and $\psi_{1}\left(q\right)=\langle q|1\rangle$ are the harmonic-oscillator eigenfunctions. The two integrals in Equation (A26) are precisely $W_{0}$ and $W_{1}$.

### Appendix C.2. Harmonic-Oscillator Wavefunctions

Let

(A27) $$\ell =\sqrt{\frac{\hbar }{m\omega_{m}}}$$

denote the oscillator length of the CNT flexural mode, and *m* its effective mass. The normalized ground-state and first-excited-state wavefunctions are

(A28) $$\begin{array}{cc} \psi_{0}\left(q\right) & =\frac{1}{{\left(\pi \ell^{2}\right)}^{1/4}}\exp\;\left(-\frac{q^{2}}{2\ell^{2}}\right), \end{array}$$

(A29) $$\begin{array}{cc} \psi_{1}\left(q\right) & =\frac{\sqrt{2}\;q}{\ell {\left(\pi \ell^{2}\right)}^{1/4}}\exp\;\left(-\frac{q^{2}}{2\ell^{2}}\right). \end{array}$$

### Appendix C.3. Ground-State Wigner Function W_*0*_

Substituting $\rho_{m}=\left|0\rangle \langle 0\right|$ into Equation (A25) gives

(A30) $$W_{0}\left(q,p\right)=\frac{1}{2\pi \hbar }\int_{-\infty }^{\infty }d\xi \;e^{-ip\xi /\hbar }\psi_{0}\;\left(q+\frac{\xi }{2}\right)\psi_{0}^{*}\;\left(q-\frac{\xi }{2}\right).$$

Using Equation (A28),

(A31) $$\begin{array}{cc} \psi_{0}\;\left(q+\frac{\xi }{2}\right)\psi_{0}^{*}\;\left(q-\frac{\xi }{2}\right) & =\frac{1}{\sqrt{\pi }\;\ell }\exp\;\left[-\frac{{\left(q+\xi /2\right)}^{2}+{\left(q-\xi /2\right)}^{2}}{2\ell^{2}}\right]\\ \; & =\frac{1}{\sqrt{\pi }\;\ell }\exp\;\left(-\frac{q^{2}}{\ell^{2}}-\frac{\xi^{2}}{4\ell^{2}}\right). \end{array}$$

Therefore

(A32) $$W_{0}\left(q,p\right)=\frac{e^{-q^{2}/\ell^{2}}}{2\pi \hbar \;\sqrt{\pi }\;\ell }\int_{-\infty }^{\infty }d\xi \;\exp\;\left(-\frac{\xi^{2}}{4\ell^{2}}-\frac{ip\xi }{\hbar }\right).$$

Evaluating the Gaussian integral yields

(A33) $$W_{0}\left(q,p\right)=\frac{1}{\pi \hbar }\exp\;\left(-\frac{q^{2}}{\ell^{2}}-\frac{\ell^{2}p^{2}}{\hbar^{2}}\right).$$

### Appendix C.4. First-Excited-State Wigner Function W_*1*_

For the one-phonon state,

(A34) $$W_{1}\left(q,p\right)=\frac{1}{2\pi \hbar }\int_{-\infty }^{\infty }d\xi \;e^{-ip\xi /\hbar }\psi_{1}\;\left(q+\frac{\xi }{2}\right)\psi_{1}^{*}\;\left(q-\frac{\xi }{2}\right).$$

Using Equation (A29),

(A35) $$\begin{array}{cc} \psi_{1}\;\left(q+\frac{\xi }{2}\right)\psi_{1}^{*}\;\left(q-\frac{\xi }{2}\right) & =\frac{2}{\ell^{2}}\left(q+\frac{\xi }{2}\right)\left(q-\frac{\xi }{2}\right)\psi_{0}\;\left(q+\frac{\xi }{2}\right)\psi_{0}^{*}\;\left(q-\frac{\xi }{2}\right)\\ \; & =\frac{2}{\ell^{2}}\left(q^{2}-\frac{\xi^{2}}{4}\right)\frac{1}{\sqrt{\pi }\;\ell }\exp\;\left(-\frac{q^{2}}{\ell^{2}}-\frac{\xi^{2}}{4\ell^{2}}\right). \end{array}$$

Substituting into Equation (A34) gives

(A36) $$W_{1}\left(q,p\right)=\frac{2\;e^{-q^{2}/\ell^{2}}}{2\pi \hbar \;\ell^{2}\sqrt{\pi }\;\ell }\int_{-\infty }^{\infty }d\xi \;\left(q^{2}-\frac{\xi^{2}}{4}\right)\exp\;\left(-\frac{\xi^{2}}{4\ell^{2}}-\frac{ip\xi }{\hbar }\right).$$

Carrying out the Gaussian integrals yields

(A37) $$W_{1}\left(q,p\right)=\frac{1}{\pi \hbar }\left[2\left(\frac{q^{2}}{\ell^{2}}+\frac{\ell^{2}p^{2}}{\hbar^{2}}\right)-1\right]\exp\;\left(-\frac{q^{2}}{\ell^{2}}-\frac{\ell^{2}p^{2}}{\hbar^{2}}\right).$$

### Appendix C.5. Final Form of the Reduced Mechanical Wigner Function

Substituting Equations (A33) and (A37) into Equation (A24) gives

(A38) $$\begin{array}{cc} W_{m}\left(q,p,t\right) & =\cos^{2}\left(gt\right)\;\frac{1}{\pi \hbar }\exp\;\left(-\frac{q^{2}}{\ell^{2}}-\frac{\ell^{2}p^{2}}{\hbar^{2}}\right)\\ \; & \;+\sin^{2}\left(gt\right)\;\frac{1}{\pi \hbar }\left[2\left(\frac{q^{2}}{\ell^{2}}+\frac{\ell^{2}p^{2}}{\hbar^{2}}\right)-1\right]\exp\;\left(-\frac{q^{2}}{\ell^{2}}-\frac{\ell^{2}p^{2}}{\hbar^{2}}\right). \end{array}$$

Equivalently,

(A39) $$W_{m}\left(q,p,t\right)=\cos^{2}\left(gt\right)\;W_{0}\left(q,p\right)+\sin^{2}\left(gt\right)\;W_{1}\left(q,p\right),$$

which is the expression quoted in the main text.

It is often convenient to introduce dimensionless phase-space variables

(A40) $$Q=\frac{q}{\ell },\;P=\frac{\ell p}{\hbar },$$

in terms of which

(A41) $$\begin{array}{cc} W_{0}\left(Q,P\right) & =\frac{1}{\pi \hbar }e^{-(Q^{2}+P^{2})}, \end{array}$$

(A42) $$\begin{array}{cc} W_{1}\left(Q,P\right) & =\frac{1}{\pi \hbar }\left[2(Q^{2}+P^{2})-1\right]e^{-(Q^{2}+P^{2})}. \end{array}$$

These expressions make clear that the reduced mechanical phase-space distribution oscillates between the Gaussian vacuum profile and the nonclassical one-phonon profile as the excitation is coherently exchanged between the spin and the CNT vibrational mode.
